# Supplementary material for: Identifying Bayesian optimal experiments for uncertain biochemical pathway models
Source: Sci Rep. 2024 Jul 2;14:15237. doi: 10.1038/s41598-024-65196-w (PMC11219779; doi:10.1038/s41598-024-65196-w)
Supplement: Supplementary file 1 — Supplementary Information. [file 41598_2024_65196_MOESM1_ESM.pdf]

Identifying Bayesian Optimal Experiments for Uncertain Biochemical Pathway Models

Natalie M. Isenberg*
Pacific Northwest National Laboratory†
Richland WA, 99354, USA
natalie.isenberg@pnnl.gov

Susan D. Mertins
Fredrick National Laboratory for Cancer Research
Fredrick MD, 21702, USA
susan.mertins2@nih.gov

Byung-Jun Yoon
Texas A&M University
College Station TX, 77843, USA
bjyoon@tamu.edu

Kristofer G. Reyes
University at Buffalo
Buffalo NY, 14260, USA
kreyes3@buffalo.edu

Nathan M. Urban
Brookhaven National Laboratory
Upton NY, 11973, USA
nurban@bnl.gov

May 17, 2024

Supplemental Information

1 Uncertain Parameter Information

Prior information for the uncertain parameters considered in this study was first elicited from expert understanding of the system and relevant sources and is compiled in Table 1. This table shows the model parameters, their nominal values, and estimated 90% confidence interval lower and upper limits.

Parameter	Units	Nominal Value	Lower Confidence Bound	Upper Confidence Bound
s_1	mol s^{-1}	1×10^{-2}	4.5×10^{-3}	2.2×10^{-2}
s_2	mol s^{-1}	3×10^{-2}	1.3×10^{-2}	6.7×10^{-2}
s_3	mol s^{-1}	2×10^{1}	4.2×10^{-1}	9.42×10^{2}
s_4	mol s^{-1}	2×10^{-1}	4.5×10^{-2}	8.9×10^{-1}
d_1	s^{-1}	1×10^{-3}	4.5×10^{-4}	2.2×10^{-3}
d_2	s^{-1}	1×10^{-4}	2.0×10^{-5}	5.1×10^{-4}
d_3	s^{-1}	2×10^{-4}	3.9×10^{-5}	1.0×10^{-3}
M	-	100,000	90,453	110,554
b_1	mol^{-1} s^{-1}	3×10^{-5}	3.9×10^{-6}	2.3×10^{-4}
a_1	mol^{-1} s^{-1}	2×10^{-10}	4.14×10^{-11}	9.66×10^{-10}
a_2	mol^{-1} s^{-1}	1×10^{-12}	6.55×10^{-13}	1.53×10^{-12}

Table 1: Table of uncertain parameters and 90% confidence limits for the *PARP1* inhibitor ODE model. All prior probability distributions are assumed to be log-normal to prohibit negative rate parameter values.

Derived prior probability distributions for each uncertain parameter considered in the model are described in Table 2. All priors are defined as Log-Normal probability distributions. For all parameters, the nominal value was taken as the mean. Because the lower and upper bounds on the uncertain parameters are not centered at the nominal value, the

*Corresponding author
†The work published was conducted at Brookhaven National Laboratory

width of this CI range was used to compute the geometric distance from the nominal to new lower and upper bounds in log-space.

Parameter	Log Mean	Log Std
s_1	-4.61	0.49
s_2	-3.51	0.49
s_3	3.00	2.34
s_4	-1.61	0.91
d_1	-6.91	0.49
d_2	-9.21	1.00
d_3	-8.52	1.00
M	11.51	0.06
b_1	-10.41	1.24
a_1	-22.33	0.96
a_2	-27.63	0.26

Table 2: Table of distributional information (mean, standard deviation) for Log-Normal priors on all uncertain parameters considered in this work.

2 Experimental Designs

The list of measurable species considered as potential "experimental designs" in the present study are shown in Table 3.

Species Symbol	Definition
mRNA-Bax	Messenger mRNA for Bax
Bad-Bcl-xL	Bad bound to Bcl-xL
Casp-pro	Inactive caspase
Bax-Bcl-xL	Bax bound to Bcl-xL
Casp-act	Activated caspase

Table 3: Table of measurable species in the *PARP1* inhibited ODE model. As in,[1] all initial values for these species is 0. The ODE model computes concentrations in dimensionless units *[Molec.]* representing molecules-per-cell.

3 Tabulated Objective Data

Measured Protein	σ_{apop}									
	$IC_{50} =$	0.001	0.01	0.03	0.1	0.3	1.0	3.0	10	100
mRNA-Bax		0.0	0.00446	0.0077	0.0128*	0.0190*	0.0261*	0.0231*	0.0190*	0.0
Bad-Bcl-xL		0.0	0.00446	0.0076	0.0144	0.0199	0.0277	0.0271	0.0195	0.0
Casp-pro		0.0	0.0119	0.0205	0.0355	0.0413	0.0494	0.0435	0.0273	0.0
Bax-Bcl-xL		0.0	0.0041	0.0095	0.0342	0.0353	0.0309	0.0283	0.0211	0.0
Casp-act		0.0	0.0034*	0.0074*	0.0158	0.0289	0.0421	0.0418	0.0277	0.0

Table 4: Table of σ_{apop} results for each measureable protein in the *PARP1*-inhibited cell apoptosis model. The minimum value in each column is denoted with an *.

$\sigma_{apop}(0.01)$	$\sigma_{IC_{50}}(0.9)$	$w_1 = 0.01$	$w_1 = 0.1$
ξ^* = Casp-act	ξ^* = mRNA-Bax	ξ^* = Casp-act	ξ^* = mRNA-Bax

Table 6: Table of optimal experimental designs, ξ^* for each objective considered in the present study.

4 Posterior Chain Summary Statistics

Each table in this section presents summary statistics for uncertain parameter values and effective sample size for each uncertain parameter inferred in Bayesian inference against a specific species measurement. Averages are computed over

Measured Species	$\sigma_{IC_{50}}$					
	Threshold=	0.5	0.6	0.7	0.8	0.9
mRNA-Bax		0.170*	0.113*	0.075*	0.047*	0.023*
Bad-Bcl-xL		0.179	0.117	0.078	0.049	0.026
Casp-pro		0.327	0.226	0.153	0.095	0.047
Bax-Bcl-xL		0.208	0.147	0.102	0.066	0.033
Casp-act		0.390	0.278	0.179	0.109	0.053

Table 5: Table of $\sigma_{IC_{50}}$ results for each measureable protein in the *PARP1*-inhibited cell apoptosis model. The minimum value in each column is denoted with an *.

100 synthetic experimental datum for that protein generated according to the procedure described in the STAR Methods. An additional table is provided per each species to list the min, max, and average chain size from all 100 posteriors used in obtaining these statistics.

mRNA-Bax

Parameter	Mean Value	Average ESS
s_1	1.01×10^{-2}	561
s_2	3.70×10^{-2}	565
s_3	141.3	566
s_4	2.80×10^{-1}	563
d_1	1.14×10^{-3}	560
d_2	1.33×10^{-4}	550
d_3	2.74×10^{-4}	569
M	100,276	565
b_1	6.82×10^{-5}	566
a_1	3.27×10^{-10}	562
a_2	1.03×10^{-12}	549

Table 7: Average expected parameter values and effective sample size from filtered chains for posteriors obtained via mRNA-Bax data.

Min	Max	Average
348	690	594

Table 8: Chain length statistics (min, max, and average) after sample rejection for mRNA-Bax.

Bad-Bcl-xL

Parameter	Mean Value	Average ESS
s_1	1.02×10^{-2}	549
s_2	3.74×10^{-2}	541
s_3	136.875	542
s_4	2.82×10^{-1}	540
d_1	1.12×10^{-3}	545
d_2	1.36×10^{-4}	531
d_3	2.79×10^{-4}	539
M	101,239	554
b_1	7.09×10^{-5}	525
a_1	3.30×10^{-10}	550
a_2	1.04×10^{-12}	557

Table 9: Average expected parameter values and effective sample size from filtered chains for posteriors obtained via Bad-Bcl-xL data.

Min	Max	Average
111	715	606

Table 10: Chain length statistics (min, max, and average) after sample rejection for Bad-Bcl-xL.

Casp-pro

Parameter	Mean Value	Average ESS
s_1	7.91×10^{-3}	226
s_2	2.84×10^{-2}	238
s_3	34.15	195
s_4	2.4×10^{-1}	235
d_1	7.93×10^{-4}	233
d_2	8.19×10^{-5}	228
d_3	3.07×10^{-4}	195
M	75,191	224
b_1	4.90×10^{-5}	228
a_1	2.47×10^{-10}	219
a_2	7.50×10^{-13}	220

Table 11: Average expected parameter values and effective sample size from filtered chains for posteriors obtained via Casp-pro data.

Min	Max	Average
192	1343	648

Table 12: Chain length statistics (min, max, and average) after sample rejection for Casp-pro.

Bax-Bcl-xL

Parameter	Mean Value	Average ESS
s_1	1.02×10^{-2}	623
s_2	3.73×10^{-2}	620
s_3	129.6	616
s_4	2.83×10^{-1}	614
d_1	1.10×10^{-3}	615
d_2	1.32×10^{-4}	606
d_3	2.82×10^{-4}	610
M	100,209	610
b_1	7.48×10^{-5}	606
a_1	3.25×10^{-10}	607
a_2	1.03×10^{-12}	614

Table 13: Average expected parameter values and effective sample size from filtered chains for posteriors obtained via Bax-Bcl-xL data.

Min	Max	Average
107	1115	812

Table 14: Chain length statistics (min, max, and average) after sample rejection for Bax-Bcl-xL.

Casp-act

Parameter	Mean Value	Average ESS
s_1	9.00×10^{-3}	249
s_2	3.31×10^{-2}	250
s_3	110.6	243
s_4	2.11×10^{-1}	249
d_1	1.05×10^{-3}	245
d_2	1.39×10^{-4}	247
d_3	1.80×10^{-4}	242
M	91,317	259
b_1	6.26×10^{-5}	249
a_1	2.83×10^{-10}	247
a_2	9.51×10^{-13}	253

Table 15: Average expected parameter values and effective sample size from filtered chains for posteriors obtained via Casp-act data.

Min	Max	Average
265	1568	1180

Table 16: Chain length statistics (min, max, and average) after sample rejection for Casp-act.

5 Prior predictive distributions

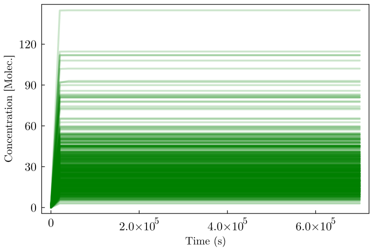

(a) mRNA-Bax

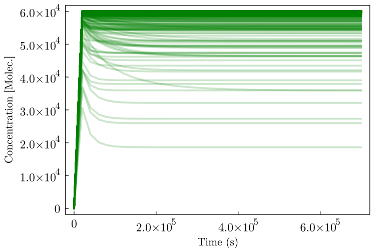

(b) Bad-Bcl-xL

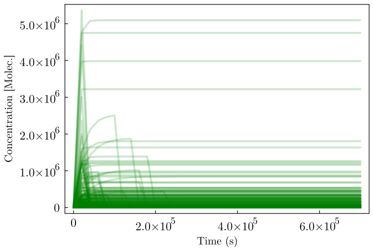

(c) Casp-pro

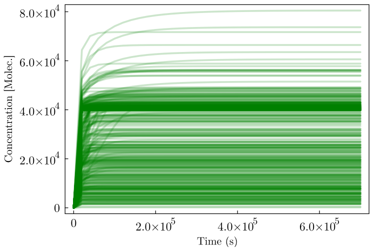

(d) Bax-Bcl-xL

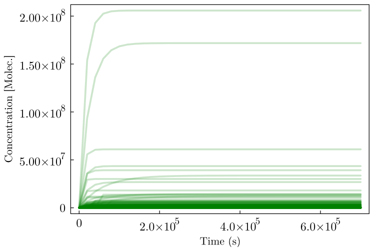

(e) Casp-act

Figure 1: Prior predictive plots from 500 simulations sampled randomly from the prior distributions of the uncertain parameters and run through the forward ODE model to create concentration profile distributions. Units of concentration are in molecules-per-cell.

6 Mathematical Model

This section contains the full system of differential equations that define the *PARP1*-inhibited cell apoptosis model as presented in.[1] In the notation in Equation 1, $\dot{x}_i$ refers to the time-varying rate of change of species i, where

$i = \{1, \ldots, 23\}$, x_i is the species concentration in units of molec/cell, and $p_i,\ i = \{1, \ldots, 27\}$ are the ODE parameters related to rates. See Tables 17 and 18 for a mapping from x_i and p_i notation to biological symbols and values. This mathematical model was generated from the rule-based modeling software, BioNetGen, using the previously published PARP1-inhibited cell apoptosis model in.[1]

$$
\begin{aligned}
\dot{x_1} &= -p_{12}x_2x_1 - p_{12}x_6x_1 + p_{16}x_{11} + p_{16}x_{12} + p_{24}x_{10}x_{12} - p_{25}x_{19}x_1 \\
\dot{x_2} &= -p_{12}x_2x_1 + p_{16}x_{11} \\
\dot{x_3} &= -p_{10}x_3x_4 - p_9x_{14}x_3 + p_{14}x_{15} + p_{42}x_{15} + p_{13}x_{20} + p_6x_{20} \\
\dot{x_4} &= -p_{10}x_3x_4 - p_{41}x_4 + p_{14}x_{15} + p_{18}x_{16} + p_{18}x_{21} \\
\dot{x_5} &= -p_{11}x_{16}x_5 + p_{15}x_{21} + p_{18}x_{21} \\
\dot{x_6} &= -p_{12}x_6x_1 + p_{16}x_{12} \\
\dot{x_7} &= -p_{21}x_{12}x_7 + p_{22}x_{18} + p_{26}x_{18}x_8 \\
\dot{x_8} &= -p_{26}x_{18}x_8 + p_{27}x_9x_{23} \\
\dot{x_9} &= 0 \\
\dot{x_{10}} &= -p_{24}x_{10}x_{12} + p_{25}x_{19}x_1 \\
\dot{x_{11}} &= +p_{12}x_2x_1 - p_{16}x_{11} \\
\dot{x_{12}} &= +p_{12}x_6x_1 - p_{16}x_{12} - p_{21}x_{12}x_7 - p_{24}x_{10}x_{12} + p_{22}x_{18} + p_{26}x_{18}x_8 + p_{25}x_{19}x_1 \\
\dot{x_{13}} &= +p_1 + p_{39} - p_5x_{13} \\
\dot{x_{14}} &= +p_{40} - p_6x_{14} - p_9x_{14}x_3 + p_{13}x_{20} \\
\dot{x_{15}} &= +p_{10}x_3x_4 - p_{14}x_{15} - p_{42}x_{15} \\
\dot{x_{16}} &= +p_{41}x_4 - p_{18}x_{16} - p_{11}x_{16}x_5 + p_{42}x_{15} + p_{15}x_{21} \\
\dot{x_{17}} &= +p_3 - p_7x_{17} - p_{43}x_{17} \\
\dot{x_{18}} &= +p_{21}x_{12}x_7 - p_{22}x_{18} - p_{26}x_{18}x_8 \\
\dot{x_{19}} &= +p_{24}x_{10}x_{12} - p_{25}x_{19}x_1 \\
\dot{x_{20}} &= +p_9x_{14}x_3 - p_{13}x_{20} - p_6x_{20} \\
\dot{x_{21}} &= +p_{11}x_{16}x_5 - p_{15}x_{21} - p_{18}x_{21} \\
\dot{x_{22}} &= +p_{43}x_{17} - p_7x_{22} \\
\dot{x_{23}} &= +p_{26}x_{18}x_8 - p_{27}x_9x_{23}
\end{aligned}
\tag{1}
$$

Symbol	Species	Description	Initial Values
x_1	DNADSB	DNA double strand break	1.74×10^5
x_2	p53	p53 transcription factor	8.5×10^4
x_3	Bcl-xL	Anti-apoptotic factor	1×10^5
x_4	Bad	Pro-apoptotic factor	6×10^4
x_5	Scaffold 14-3-3	Scaffold 14-3-3	2×10^5
x_6	PARP	Poly(ADP-ribose) polymerase	1.07×10^5
x_7	NAD	Nicotinamide adenosine deoxyribose	1.07×10^6
x_8	XRCC1(Glu∼uPAR)	PARP substrate XRCC1 unPARylated	1.07×10^5
x_9	PARG	Poly(ADP-ribose) glycohydrase	1.07×10^4
x_{10}	Inhibitor	Generic PARP inhibitor	0.005083
x_{11}	DNADSB-p53	p53 bound to DNA double strand break	0
x_{12}	DNADSB-PARP	PARP bound to DNA double strand break	0
x_{13}	mRNA-Bax	Messanger RNA for Bax	0
x_{14}	Bax	Pro-apoptotic factor	0
x_{15}	Bad-Bcl-xL	Bad bound to Bcl-xL	0
x_{16}	Bad(S75 S99∼PP,b)	Phosphorylated Bad	0
x_{17}	Casp-pro	Inactive caspase	0
x_{18}	DNADSB-NAD-PARP	NAD bound to PARP active site while PARP bound to DNA	0
x_{19}	Inh-PARP	Inhibitor bound to PARP	0
x_{20}	Bax-Bcl-xL	Bax bound to Bcl-xL	0
x_{21}	Bad-Scaffold 14-3-3	Bad bound to Scaffold 14-3-3	0
x_{22}	Casp-act	Active caspase	0
x_{23}	XRCC1(Glu∼Par)	PARP substrate XRCC1 PARylated	0

Table 17: Mapping between symbol and species name in the ODE model in Equation 1.

Symbol	Species	Nominal Value
p_1	s_1	0.01
p_2	s_2	0.03
p_3	s_3	20
p_4	s_4	0.2
p_5	d_1	0.001
p_6	d_1	0.0001
p_7	d_1	0.002
p_8	M	10,000
p_9	b_1	3×10^{-5}
p_{10}	b_2	0.003
p_{11}	b_3	0.003
p_{12}	b_4	3×10^{-5}
p_{13}	u_1	0.0001
p_{14}	u_2	0.0001
p_{15}	u_3	0.0001
p_{16}	u_4	0.0001
p_{17}	p_1	3×10^{-10}
p_{18}	q_1	3×10^{-5}
p_{19}	a_1	2×10^{-10}
p_{20}	a_2	1×10^{-12}
p_{21}	kf_1	0.001
p_{22}	kr_1	3.79
p_{23}	IC_{50}	N/A
p_{24}	kf_2	0.001
p_{25}	kr_2	0.00379
p_{26}	$kcat_1$	1
p_{27}	$kcat_2$	1

Table 18: Mapping between symbol and parameter name in the ODE model in Equation 1. There is no nominal value specified for p_{23}=IC_{50} because it is a decision variable and its value is subject to change within our studies.

References

[1] Susan D. Mertins, Natalie M. Isenberg, Kristofer-Roy Reyes, Byung-Jun Yoon, Nathan Urban, Manasi P. Jogalekar, Morgan E. Diolaiti, M. Ryan Weil, and Eric A. Stahlberg. Pharmacodynamic model of parp1 inhibition and global sensitivity analyses can lead to cancer biomarker discovery. *bioRxiv*, 2023.
